# Supplementary material for: Metagenomic binning with assembly graph embeddings
Source: Bioinformatics. 2022 Aug 16;38(19):4481–7. doi: 10.1093/bioinformatics/btac557 (PMC9525014; doi:10.1093/bioinformatics/btac557)
Supplement: btac557_supplementary_data [file btac557_supplementary_data.zip › amber outputs.zip!/amber outputs/strong100/semibin_results/genome/bin_counts.pdf]

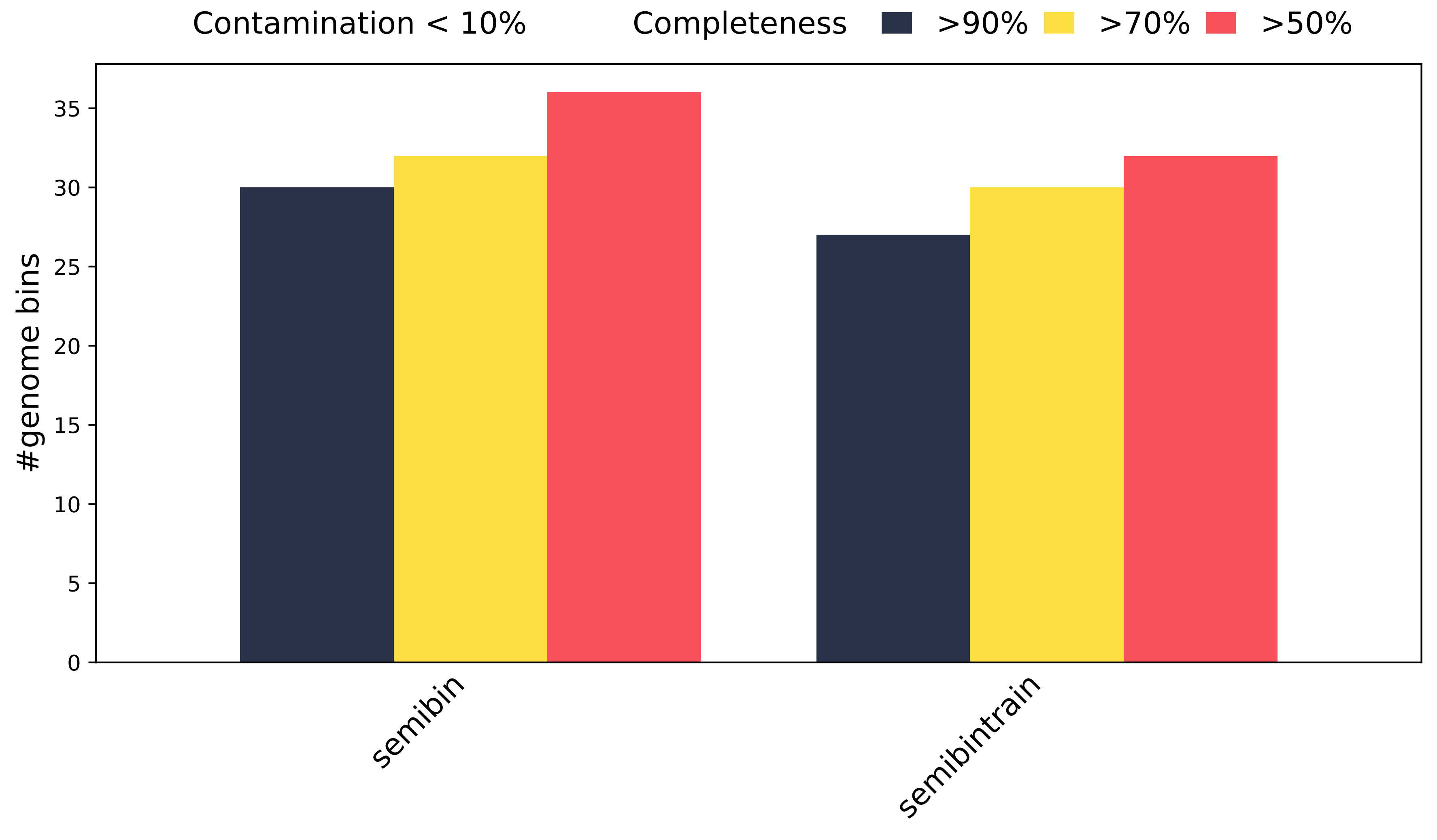
Contamination < 10%
Completeness
>90%
>70%
>50%
#genome bins
0
5
10
15
20
25
30
35
semibin
semibintrain
